# A Review on Photonic Sensing Technologies: Status and Outlook

**DOI:** 10.3390/bios13050568

**Published:** 2023-05-22

**Authors:** Muhammad A. Butt, Nikolay L. Kazanskiy, Svetlana N. Khonina, Grigory S. Voronkov, Elizaveta P. Grakhova, Ruslan V. Kutluyarov

**Affiliations:** 1 Samara National Research University, 443086 Samara, Russia; 2 IPSI RAS—Branch of the FSRC “Crystallography and Photonics” RAS, 443001 Samara, Russia; 3 Ufa University of Science and Technology, Z. Validi St. 32, 450076 Ufa, Russia

**Keywords:** photonic sensor, optic fiber, optical waveguide, photonic crystal, metasurface, plasmonics

## Abstract

In contemporary science and technology, photonic sensors are essential. They may be made to be extremely resistant to some physical parameters while also being extremely sensitive to other physical variables. Most photonic sensors may be incorporated on chips and operate with CMOS technology, making them suitable for use as extremely sensitive, compact, and affordable sensors. Photonic sensors can detect electromagnetic (EM) wave changes and convert them into an electric signal due to the photoelectric effect. Depending on the requirements, scientists have found ways to develop photonic sensors based on several interesting platforms. In this work, we extensively review the most generally utilized photonic sensors for detecting vital environmental parameters and personal health care. These sensing systems include optical waveguides, optical fibers, plasmonics, metasurfaces, and photonic crystals. Various aspects of light are used to investigate the transmission or reflection spectra of photonic sensors. In general, resonant cavity or grating-based sensor configurations that work on wavelength interrogation methods are preferred, so these sensor types are mostly presented. We believe that this paper will provide insight into the novel types of available photonic sensors.

## 1. Introduction

In the realm of optical signal development for sensing applications in many fields, particularly in chemical and biochemical detection, angular rate rotation estimation and electric field detection waveguide (WG)-based devices are becoming more and more appealing. The fascination with optical sensing is supported by unparalleled benefits made possible by photonic technologies such as high sensitivity (S), compatibility with electronic devices, compactness, metal-free operation, affordability, and EM resistance. Ring resonators (RRs) [1,2,3] and surface plasmons (SPs) [4] have recently caught the attention of scientists since they can significantly improve the effectiveness of integrated photonic sensors [5,6]. Photonic sensors built on fiber and WG technology have drawn much attention because they have a broad range of possible uses [7]. Because they are immune to EM fields, optical sensors function better than other types of sensors, especially in severe conditions like those found in electrical power generating and conversion plants. Distributed fiber sensors open new possibilities for monitoring cables, pipelines, and locations that require high levels of security. Additionally, optical biosensors are becoming more and more crucial, for instance, in solutions for labs-on-chips in health care [8].

By effectively transforming the bio-entity into an electrical form that can be studied using a spectrometer, photonic sensors are designed to detect a range of bio analytes [9]. It is possible to distinguish between normal and cancerous cells using electrical properties and examining the electric field [10]. In addition, the refractive indices of different analytes become an important factor in creating accurate biosensors [11,12,13]. Label-free biosensors are a potential type of biomolecular detector since they do not require a fluorescent, radio, or enzymatic label. Depending on such a label to recognize a biomolecular interaction might negatively influence the sensing performance, either by interacting with the binding event or by non-specific adsorption of the labeling molecule [14]. There are several well-developed approaches for direct label-free detection of bound target biomolecules, comprising optical [15,16], electrical [17], and acoustic sensing devices [18]. Due to their ability for multiplexed detections, ability to work in aquatic conditions, and capacity to focus EM energy into tiny mode volumes, optically resonant devices are gaining significance within the larger class of label-free sensing tools. To create optically resonant biosensors, a variety of architectural designs such as photonic crystals (PCs) [19], microtoroids [20], and ring/racetracks [1,21,22] have been studied.

Liquid biopsy is a non-invasive technique used to detect and analyze biomarkers in a patient’s bodily fluids such as blood, urine, or cerebrospinal fluid [23]. It offers several advantages over traditional tissue biopsies as it provides real-time and dynamic information about a patient’s condition, enables monitoring of disease progression or treatment response, and can be performed repeatedly with minimal discomfort to the patient. Liquid biopsy can benefit from the use of photonic sensors, which leverage the principles of optics and photonics to detect and analyze biomarkers in bodily fluids [24]. For instance, surface plasmon resonance (SPR) can be employed to detect biomarkers by immobilizing specific capture molecules such as antibodies or aptamers onto a sensor surface. When target biomarkers bind to the capture molecules, it causes a shift in the SPR signal, enabling their detection and quantification. PC-based sensors can be functionalized with specific biomolecule receptors to capture and detect target biomarkers in liquid biopsy samples. Binding events between the biomarkers and the receptor molecules cause changes in the sensor’s optical properties, enabling sensitive detection. Optical fiber sensors are versatile sensing platforms that can be used for various applications including liquid biopsy. Functionalized optical fibers can also be used to selectively capture biomarkers from the sample. Changes in the refractive index or fluorescence properties of the captured biomarkers can be measured using light propagation within the fiber, enabling their detection and analysis [25].

The study of nonlinear optics and machine learning techniques provides a comprehensive overview of optical biosensors that can be enhanced [26]. A wide variety of viruses have been successfully detected by optical biosensors. In particular, the SARS-CoV-2 virus has caused havoc throughout the world, and biosensors have become essential for offering an analysis based on physical and chemical phenomena. In this view, a multiphoton interaction that may be the cause of the increased sensitivity displayed by biosensors has been examined. The nonlinear optical effects give rise to several possibilities for expanding the uses of optical biosensors. Computer techniques and nonlinearities work well together to identify complicated low-dimensional agents. The detection of dynamic objects inside the human body and the identification of viruses, dangerous organisms, and unusual kinetics in cells are two examples of how machine-learning techniques may approximate functions to uncover patterns [26].

**Figure 1:**
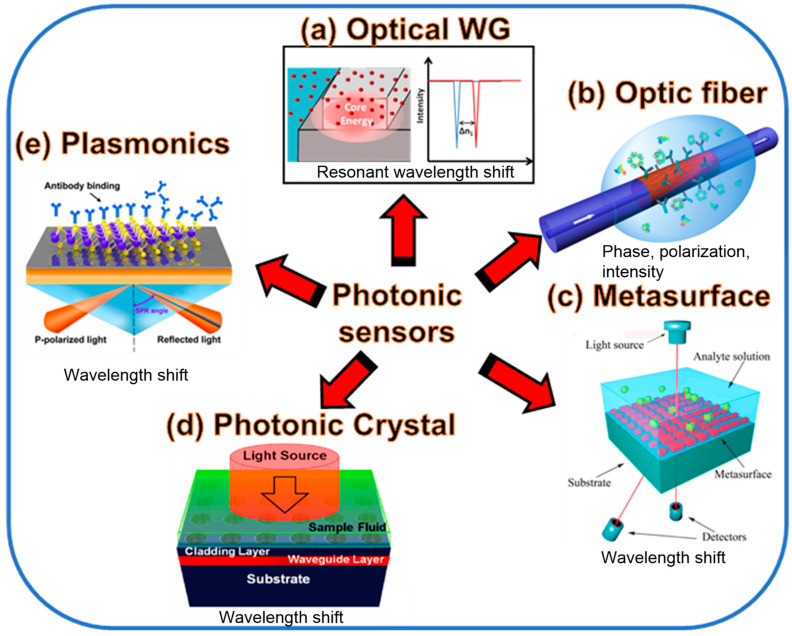
The photonic sensing technologies based on (**a**) optical WG [28], (**b**) OF [29], (**c**) MS [30], (**d**) PC [31], and (**e**) plasmonics [32] discussed in this paper.

In this paper, recent advancements in the field of photonic sensors based on optical WGs, optical fibers (OFs), metasurfaces (MSs), PCs, and plasmonics are thoroughly discussed for numerous sensing applications (Figure 1). These sensors are highly appealing due to their compact size and high sensitivity. These sensors can register various changes in the parameters of optical radiation (phase, polarization) with environmental changes [27]. For example, Rayleigh scattering affects radiation modes polarized along the x and y axes differently, which leads to a change in the polarization of light in the fiber. Change in the permittivity of the environment or optical propagation medium due to external influences for example. The KerrA effect leads to a change in the optical length of the fiber and a change in phase shifts. In Section 2, recent advances in photonic sensors based on WGs are discussed. Several novel WG designs are investigated to enhance the S of the photonic sensors. Section 3 discusses the progress in OF-based sensors, which can be utilized for diverse applications including biochemical sensing and environmental monitoring. PC-based fibers are susceptible to the ambient medium, which opens the way for advancing highly sensitive fiber-based sensors. Section 4 discusses the recent developments in PC WG-based sensors. The application of PCs in photonic sensor design is extensive. One can identify any physical processes such as temperature, pressure, strain, and the presence of chemicals and biomolecules that can alter the periodicity and refractive index (RI) of the formation of the PC by monitoring optical properties like the spectral trend of reflected and transmitted power. Over the past 20 years, metamaterials have attracted much interest because of their outstanding EM features. The MS’s transient reaction is necessary for applications in modern science and technology, but the traditional MS’s functionality is restricted in terms of tuning and customization. The structure, shape, and topology of the meta-atoms in conventional MSs normally govern their static, preset optical capabilities. In Section 5, the recent advances in MS-based photonic sensors are discussed. Last but not the least, plasmonic sensors are currently a hot topic, which has fascinated researchers to develop eye-catching and highly sensitive plasmonic devices. In Section 6, the plasmonic sensors established on a metal-insulator-metal (MIM) WG are thoroughly discussed. The paper ends with concluding remarks, as mentioned in Section 7.

## 2. Optical Waveguide-Based Sensors

Devices that can track changes in light speed include optical WGs made of various materials [33]. Nowadays, the production of silicon (Si) WGs can be done affordably and effectively with the help of silicon foundries and modern technologies [34]. In this domain, it may make sense to detect various materials while taking different WG designs into account such as the buried channel WG [35], slot WG [36], and rib WG [37]. A good approach to this issue is to consider how this technology may also be useful for communication systems and generating THz. Future research for this study might include optical WG and quantum computing [38]. Using optical Si WGs in this field is crucial, as demonstrated by recent work with Intel on quantum computers [39]. Thanks to optical memory, computers will soon be able to speed up even more and circumvent Moore’s law [40]. The WG design is one of the intriguing concepts in optical WGs. Another aspect that can enhance the device’s functionality is WG structural optimization. For this kind of study, simulation and manufacturing techniques are essential because they allow for time and cost savings while providing a more accurate picture of real devices.

The appropriate selection of operational wavelength plays a crucial role in biosensing, particularly in techniques such as optical sensing and spectroscopy [41]. Biomolecules such as proteins, DNA, and other cellular components exhibit specific absorption, reflection, or scattering properties at certain wavelengths of light. By selecting an appropriate wavelength, biosensors can target specific biomolecules and analyze their behavior or presence in a sample. The choice of wavelength can significantly affect the sensitivity and selectivity of biosensors. Different biomolecules have characteristic absorption or fluorescence spectra, and by using appropriate wavelengths, biosensors can detect and distinguish specific targets from complex samples.

**Figure 2:**
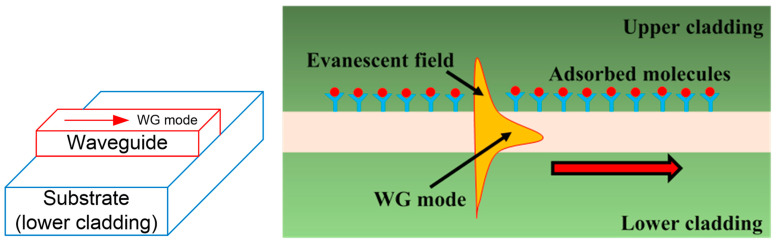
A WG evanescent field sensor is shown the an image. At the WG surface, receptor molecules catch molecular targets, modifying the WG mode effective index. As a result, the propagating optical mode is thus phase-shifted.

Si photonic biosensors that use the SOI platform detect molecular contact events using near-infrared light constrained in an optical WG. The evanescent field, or fraction of the E-field of the light that travels beyond the WG, can interplay with the adjacent volume to form an external RI-sensitive zone [42]. The aggregation of molecules with various refractive indices modifies the exterior RI. It disturbs the evanescent field when target molecules connect to receptors at the surface of the WG, which then affects how guided light behaves inside the WG, as shown in Figure 2. Analytes of concern can be found instantly by observing the outgoing light’s coupling and/or propagation characteristics [43]. Since the evanescent field decays linearly into the bulk medium over a distance of a few tens to a few hundreds of nanometers, the sensing signal of an analyte collected within the decay length differs noticeably from the signal of an analyte drifting far from the surface. Thus, depending on the evanescent field sensor’s response, we can discriminate between the target molecules still in bulk solution (bulk sensing) and those trapped on the surface (surface sensing). Moreover, optical WGs based on SOI platforms offer an elegant alternative for detecting trace gases that utilize evanescent field absorption sensing. Gas sensors that rely on evanescent field absorption can only be used when the gas being monitored exhibits the expected absorption line at the appropriate wavelength. The optical attenuation at a certain wavelength and the gas concentration are also related. Several gas sensors built on OF [44,45] and WGs [37,46,47] have been suggested to function on this phenomenon.

**Figure 3:**
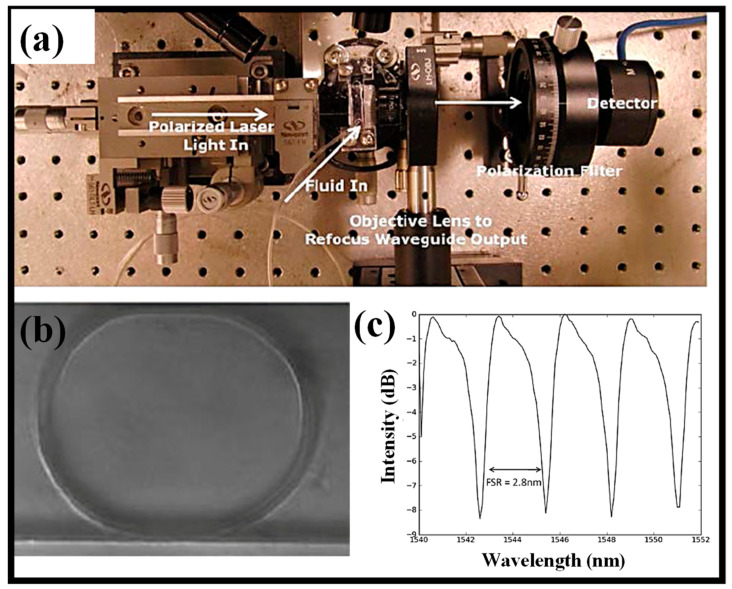
(**a**) Experimental setup to characterize the sensing device, (**b**) ring resonator device, (**c**) a standard output spectrum of a porous ring resonator. Adapted with permission from [28].

Due to their capability for multiplexed detection and their capacity to concentrate EM energy into tiny mode volumes, optically resonant devices hold promise as label-free biomolecular sensors. The fact that biomolecular interactions are restricted to the resonant device’s surface and the strongest EM energy is confined inside the core is a basic constraint of current optical biosensor technology. Ring resonator (RR)-based nanoporous polymer optofluidic devices indicate a 40% improvement in polymer device S that is ascribed to the surge in light–matter interactions [28]. To couple light into polymeric WGs, an Ando AQ4321D laser source (tunable between 1520 nm and 1620 nm) was employed. The chip and fiber are positioned on 3-axis stages to guarantee precise alignment. To avoid picking up dispersed light, the WG’s input and output facets are moved inward by 3 mm. Figure 3a illustrates how light entering the WG is gathered, collimated, and then routed via a polarizer to only choose the TM mode before arriving at the photodetector [28]. A RR imaged through the device and covered in an aqueous solution is shown in Figure 3b [28]. Figure 3c displays a standard spectrum from the RRs. Water serves as the cladding liquid, and a 1 mW laser pulse is connected to the WG. Roughly 40–100 microwatts of electricity are gathered at the detector after coupling and scattering losses. Extinctions between 3 and 15 dB at the resonant wavelength and Q-factors between 1000 and 3000 are seen, relying on the fabrication perfection of the RR [28].

**Figure 4:**
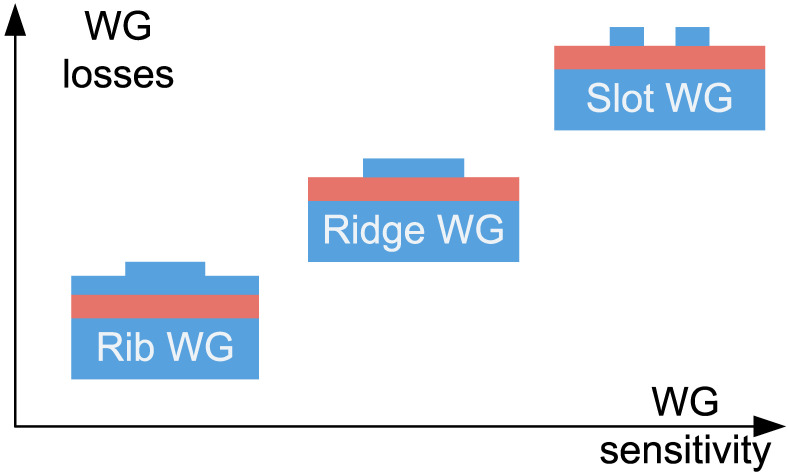
Widely utilized SOI WG structures for optical biosensing.

In the preceding ten years, several analytical research has been carried out to improve WG geometries for optical sensing [48,49]. Figure 4 illustrates the three predominant types of WGs that are commonly used. These consist of slot WGs, strip WGs, and rib WGs [50,51,52]. The top cladding material, which contains the analyte, is being substantially penetrated by the guided mode’s evanescent field. The amount of light that enters the upper cladding of each WG structure varies, and this variation correlates to unwanted optical losses; the more light that enters the upper cladding, the bigger the optical losses through absorption and scattering. Light is mostly constrained inside the high index Si core of WG structures like strips and ribs, but in slot WG design, light can be significantly trapped in the subwavelength low index medium sandwiched between two Si rails. Slot WGs are far more sensitive than ridge WGs because there is more spatial interaction between the evanescent and sensing environments. Slot WGs are hence a well-liked option for bulk index sensing. A proper WG type must be chosen according to the situation. Low optical losses are attained at the cost of S of the rib WG. Conversely, slot WGs have excellent S but a considerable optical loss. As demonstrated in Figure 4, ridge WGs, on the other hand, offer a superb combination of loss and S. The S of the WG typically increases along with the strength of the light–matter interaction, although optical losses also increase. Table 1 presents some novel works on photonic sensors established on different WG components.

Another way of developing SOI-based RR sensor systems includes structures with Bragg gratings (BGs) and slot WGs [53,54,55,56]. The interest in grating structures is caused by the desire to increase the interaction zone of the ring with the analyzed substance (weakly manifested in the standard strip RR), which allows for increasing the S of the sensor [57]. The periodically corrugated WG region can be used as a light coupling area, which is very sensitive to changes in the RI of the background environment, which can be expressed in a shift of the resonant wavelength in the spectrum. There are a variety of combinations of grating structures with μ-RRs: BG-based RR with directional WG [58]; slot directional WGs with slot RR and BGs [59]; SWG racetrack RR [57]. The combination of RR and BG structures reduces fabrication tolerances and environmental perturbations on the resulting characteristic, which occurs in the resonant structure [58].

**Table 1:** Recently proposed photonic sensors established on different optical WG components.

Device Type	Experiment/ Simulation	Sensitivity	FOM	Q-Factor	LOD	Ref.
RR established on ridge WG	Experiment	112 nm/RIU	-	-	1.6 × 10^−6^	[60]
PC heterostructure cavities	Experiment	1500 nm/RIU	-		7.8 × 10^−6^	[61]
RR established on ridge WG	Experiment	2169 nm/RIU	-	-	8.3 × 10^−6^	[62]
2D PC microcavity	Experiment	200 nm/RIU	-	400	0.002	[63]
RR established on ridge WG	Simulation	167 nm/RIU	49.9	561.6	2.75 × 10^−2^	[64]
PC slot microcavity	Experiment	370 nm/RIU	-	7500	2.3 × 10^−5^	[65]
RR established on slot WG	Experiment	-	-	-	5 × 10^−6^	[66]
RR established on slot WG	Experiment	298 nm/RIU	-	-	4.2 × 10^−5^	[67]
Mach–Zehnder interferometer	Experiment	2.5 pm/K	-	-	-	[68]
RR established on SWG double slot WG	Simulation	840 nm/RIU	6461.5	9246.2	-	[1]
Grating sensor	Experiment	1606.2 nm/RIU	-	-	3 × 10^−5^	[69]
PC point defect resonant cavity	Simulation	330 nm/RIU	-	3820	0.001	[70]
Young Interferometer	Experiment	2.2 rad/°C	-	-	6.4 × 10^−6^	[71]
PC ring-slot structure	Simulation	160 nm/RIU	-	10^7^	8.75 × 10^−5^	[72]
RR established on SWG hybrid plasmonic WG	Simulation	1000 nm/RIU	287.35	441.05	-	[73]
Young interferometer	Experiment	0.051	-	-	1 × 10^−6^	[74]
RR coupled phase-shifted BG resonator	Simulation	297.13 nm/RIU	-	2000	1.1 × 10^−4^	[59]
Slot RR and BG	Simulation	211.43 nm/RIU	-	1720	1.26 × 10^−3^	[75]
RR established on SWG	Simulation	7061 nm/RIU	-		1.74 × 10^−5^	[57]
RR established on SWG	Experiment	2659 nm/RIU (3.76 × 10^−4^ RIU/nm)	-	2.72 × 10^5^ at 1587 nm	-	[76]

## 3. Optical Fiber-Based Sensors

Utilizing the concept of total internal reflection, OFs allow for the correlation of the light intensity measured at the detector with the original target concentration [77]. To interact with the target analyte, bio-receptors such as oligonucleotides, antibodies, and enzymes can be mounted on the core surface of the fiber. Following the creation of a standard reference curve, this interplay will affect the sensitive layer’s characteristics and be correlated with the analyte concentration. Fiber-optic biosensors have the benefits of high S, resilience, durability, quick detection, high S, and real-time surveillance and are immune to EM interference [78]. These characteristics help OF biosensors work well because they can simultaneously and discretely transmit light of several wavelengths. They may be employed for multiple analyte detection employing numerous DNA probes [79]. They can be carried out label-free or label-based and can be integrated on a single chip. OF sensors come in a wide range of configurations. There are many options for OF sensors to detect different physical, chemical, and biological factors since the optical properties of most materials are inherently sensitive to their environment.

A uniform WG with periodic RI fluctuations running across it is known as a BG structure. Because of these irregularities, any broadband signal passing through the WG will only reflect a portion of its spectrum in a 1D-photonic bandgap. Intuitively, BG WGs are similar to the well-established fiber Bragg gratings (FBGs). For almost 30 years, lasers have been used to create FBGs, which are narrowband mirrors built into OFs that are frequently used for WDM adjustable filtering, and—when chirped—dispersion compensation in optical communications systems. These systems are frequently used in sensing areas since their resonant (reflected) wavelength is very receptive to environmental variables like temperature [80], RI [81,82], and strain [83]. In 1978, Ken Hill made the discovery of FBG at the Canadian Communication Research Center [84,85]. Since their creation, grating patterns have attracted a lot of attention in the field of optical sensing due to their great qualities including their affordable, small size, real-time reaction, high accuracy, high sensitivity, and EM interference. It is feasible to measure several properties such as temperature, pressure, tension, and RI utilizing grating-based devices. Today, FBGs are used in a wide variety of fields including high-temperature sensors, medical and biological devices, harsh environments, structural engineering, the oil industry, radioactive settings, and aircraft, marine, and civil engineering [86,87,88]. Since the effective index of the majority of glass materials is close to 1.5, the Bragg response in the telecom band at 1550 nm necessitates a brief grating period of around 500 nm. LPG, EFBG, tilted FBG, microstructured FBG, PC fibers, LPG inscribed in PCF, and tilted FBG coupled with SPR are a few illustrations of OF grating-based biosensors that are documented to function following diverse operating principles. The evolution of chemo- and biosensors is increasingly dependent on optical grating sensors such as LPG, EFBG, and tilted FBG sensing apparatuses due to their label-free RI measuring characteristics. Some of these FBG biosensor concepts have been investigated for thrombin biosensor development [89,90,91].

**Figure 5:**
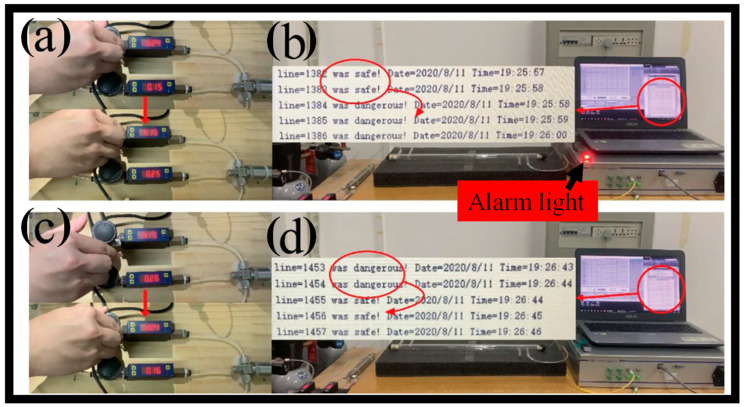
(**a**) Snapshot of the CO_2_ volume percentage in the gas chamber rising [92], (**b**) when the CO_2_ percentage in the gas chamber is over the threshold, the warning light turns on, and the output signal changes from “safe” to “dangerous” [92], (**c**) Image of the gas chamber with the CO_2_ conc. reduced [92], (**d**) when the CO_2_ conc. in the gas compartment is below the threshold, the warning light turns off, and the signal that was previously outputted as “dangerous” changes to “safe” [92].

The evolution of carbon dioxide (CO_2_) detection is crucial for the preservation of the environment. To overcome this difficulty, a novel polyether sulfone (PES)-coated FBG sensor is presented [92]. When exposed to CO_2_, the PES coating displays volume dilatation and can transmit stress to the grating, changing the grating’s period and RI. The low-temperature spin coating and high-temperature curing processes are used to provide a standardized and homogeneous PES coating, which is essential for the reproducibility and durability of the sensing device. Investigations were conducted on the effectiveness of the FBG sensor and its influencing elements. The greatest Bragg wavelength shift was inversely correlated with temperature and highly associated with coating thickness. The PES-coated FBG sensor had a minimum reaction time of 3.27 min and exhibited strong selectivity to CO_2_. For CO_2_ detection, the LOD can be as low as 0.78%. Eventually, a system for over-conc. alerts was created for online CO_2_ monitoring. The PES-coated FBG sensor’s exceptional qualities, together with its inexpensive and straightforward construction technique, open a wide range of application possibilities. An over-conc. alert system was created to be used with the PES-coated FBG sensing device in online checking systems as shown in Figure 5a–d [92]. The apparatus continually records the current value of the wavelength for each group after receiving the initial wavelength values for the experimental group and the control group. When the limit is exceeded, the created software raises an alert and turns on the warning light when the change in Bragg wavelength shift between the experimental group and the control group is larger.

As intriguing alternatives to the most conventional ones established on SPR or interferometric setups, OF gratings are being presented more commonly as optical platforms for label-free biosensing [93,94]. The effectiveness of OF gratings is comparable to that of more traditional optical platforms but with the inherent benefits of OFs including exhibiting a significant and prospective compact size, high compatibility with optoelectronic devices (both sources and detectors), and finally, multiplexing and remote measurement possibility because the signal is spectrally modulated. Traditional prism-coupling-based SPR sensing apparatuses come in two forms: Kretschmann [95] and Otto [96] arrangements. These sensor designs are established on attenuated total reflection as their underlying operating principle. Kretschmann-based SPR pattern devices are widely employed in sensor applications due to their exceptional performance, although they are subject to several limitations. These instruments are frequently large and made with moving parts. As a result, they cannot be utilized for remote monitoring or other portable applications. Additionally, spectral-based measurements are costly to apply realistically, and scaling down the sensor size is less likely. To effectively handle these upcoming difficulties, OF-based SPRs have been implemented. The OFs are compact and inexpensive. Total internal reflection underlies the transmission of light via OFs, and a SPR sensor configuration established on OFs offers several advantages over one established on prisms [97]. Additionally, the OF’s compactness allows for a considerable reduction in the size of the sensor that might be employed for remote sensing purposes. OF-based SPR sensing apparatuses provide a larger dynamic range for recognition and higher resolution but are only useful for constrained acceptance angles [98]. Numerous OF SPR sensing apparatuses have been identified in theoretical and experimental research [99,100].

Jorgenson et al. proposed the first OF-based SPP setup without the bulk prism in 1993 [101]. The interplay of evanescent waves with SPPs was used to show an OF-based SPP RI sensor. The fiber cladding was partially removed, and a highly reflective coating was applied to the exposed area. The transmission or reflection properties of the light propagating are often the basis for the operating mechanism of the plasmonic sensing apparatuses produced on OFs [102]. Noble metal and immobilized ligands are used in transmission probe-based OF sensing apparatuses to detect unidentified analytes [103]. In contrast, the backlight is reflected to the fiber by a mirror in sensing apparatuses established on a reflection probe. With noble metals assembled on the engraved cladding section of the transmission probe, a variety of fiber-optic plasmonic sensors have previously been investigated. These include single-mode fibers (SMFs) [104], multi-mode fibers (MMFs) [105], wagon wheel fibers [106], U-shaped fibers [107,108], D-shaped fibers [109,110], and FBGs [111], among others [112,113].

**Figure 6:**
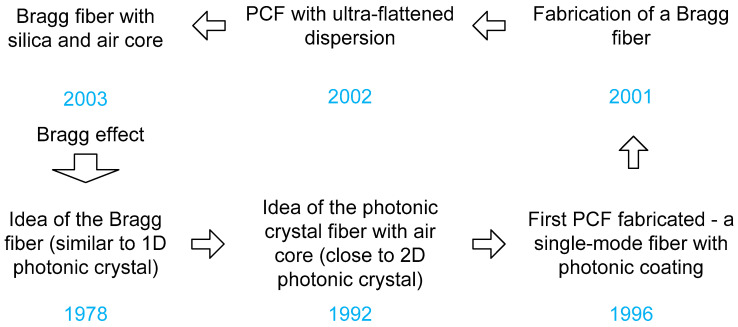
Synopsis of PCF expansion.

In 1978, the photonic crystal fiber (PCF) idea was initially proposed. A comparable idea to 1D-PC was to clad a fiber core with BG. A 2D-PC with an air core-based PCF was previously suggested in 1992, and it was disclosed at the Optical Fiber Conference (OFC) in 1996. Figure 6 summarizes the evolution of PCF. Similar to a standard OF, PCFs include a core and cladding, but they also have periodic air-holes in the cladding area that control light transmission. By adjusting the air hole geometries and ring counts, it is feasible to control how light propagates. Recently, researchers have analyzed the pattern of the E-field in a straightforward 2D PCF structure to analyze the sensing of malignant cells. Human immortalized normal oral keratinocytes, which belong to the category of normal cells, and YD-10B cells, which are malignant, were both regarded to be clusters of cell lines [114]. A new study used a SPR-based PCF biosensor construction to look at early cancer cell detection [115]. Both spectral interrogation and amplitude techniques are used to identify the RI variations of cancer cells. Based on the difference in RI between healthy and malignant blood cells, a twin-core PCF is suggested for the early diagnosis of blood cancer [116]. The middle air hole has been penetrated by the samples. The suggested biosensor’s changes in coupling length and transmitted spectrum for normal and cancerous cells have also been studied. For the identification of cancer cells in the cervical, breast, and basal regions, dual-core PCFs have been proposed [117,118,119].

**Figure 7:**
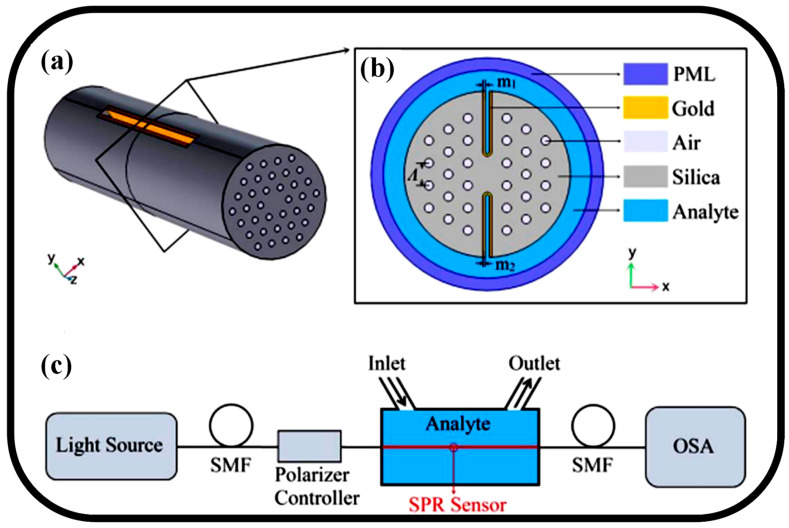
(**a**) H-shaped PCF-SPR sensor schematic diagram [122], (**b**) SPR sensor in cross-section [122], (**c**) experimental SPR sensor configuration for detecting RI [122].

PCF-based SPR sensors are more effective in SPR sensing applications thanks to their benefits of compactness, high S, and multi-parameter analysis [120]. Nevertheless, there are currently two main issues with PCF-based SPR sensors. The first issue is the challenging fabrication process, which includes metal coating and analyte loading. These sensors have very tiny holes, often to the order of micrometers [120]. As a result, loading them with an aqueous analyte within the required limits and evenly covering them with a metal layer is challenging. The limited RI region of the sensor detection, caused by either using a low RI or high RI PCF-SPR sensors, impedes the ability to utilize it for the substitution of the analyte [121]. An H-shaped SPR sensor established on PCF is presented for sensing a wide RI range, which may be either higher or lower than the RI of the fiber material utilized [122], as shown in Figure 7a. In contrast to previous models, the H-shaped PCF grooves, which serve as the sensing channels, are treated with a gold film before being introduced into direct contact with the analyte. This decreases the complexities of manufacturing and increases reuse capacity. The cross-sectional view of the SPR sensor is shown in Figure 7b. According to numerical data, the sensor can operate normally in the vast analyte RI range of 1.33 to 1.49, and it can attain its high S of 25,900 nm/RIU at the RI range of 1.47 to 1.48. Additionally, the sensor exhibits high stability within tolerances of 10% of the gold-film thickness [122]. The experimental setup that can be used to characterize the sensor is shown in Figure 7c. Table 2 presents some recent works on OF-based sensors for several sensing applications.

## 4. Photonic Crystal-Based Sensors

**Figure 8:**
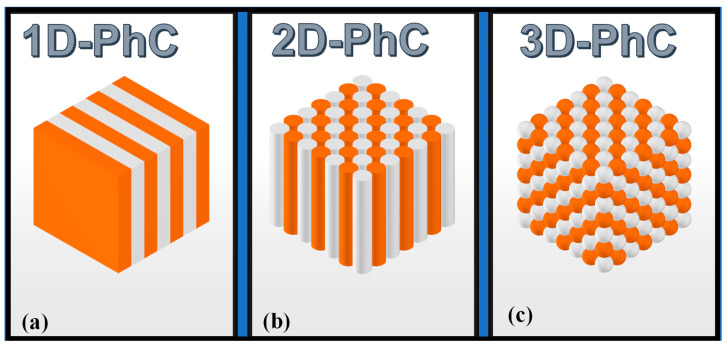
Schematic of PC formation, (**a**) 1D, (**b**) 2D, (**c**) 3D.

PCs are formations with a periodic fluctuation in one, two, or all three orthogonal directions of the dielectric constant (RI). The three types are referred to as one (1D), two (2D), and three-dimensional (3D) PCs, respectively. Multilayer architectures, or 1D-PCs, have undergone extensive study and have been documented in the literature [136]. They are made of two materials with differing refractive indices that are alternately layered, resulting in a RI that periodically varies in one direction but is homogenous in the other two. When the RI varies in two directions but not the third, the crystal is said to be a 2D-PC [137,138,139]. This may be carried out by arranging cylinders of any dielectric material in the air or by drilling holes with triangular or square symmetry in a substance with a high RI. The RI of 3D-PCs is altered in all three spatial directions, for example, by stacking spheres of a dielectric substance in the air [140]. The schematic of the 1D, 2D, and 3D PC formations is shown in Figure 8.

Concerning the CMOS-compatible silicon-on-insulator (SOI) technology, the advancement and integration of microfluidic and photonic innovation and technology for the improvement of sensing performance in terms of S, limit-of-detection (LOD), and detection multiplexing potential have been studied [141,142,143]. Over the past 10 years, photonic sensors have been the focus of many studies, particularly for the recognition of a wide range of biological and chemical substances. Since they are anticipated to have higher S and selectivity in addition to high stability, immunity to EM interference, and quality enhancement such as smaller integration sizes and lower costs, photonic lab-on-a-chip systems represent the latest in photonic sensing in this perspective. Designing photonic sensors makes extensive use of PCs. By monitoring optical properties like the spectral trend of reflected and transmitted power, one can detect any physical processes such as temperature, pressure, strain, and the occurrence of chemicals and biomolecules that can change the periodicity and RI of the formation of the PC [144].

PCs have excellent optical properties that restrict light to a very tiny volume, making it possible to identify chemical species with nanometer-sized molecules [145]. Additionally, very good efficiency in ultra-compact sensor chips may be produced by integrating modern chemical surface functionalization processes with microfluidic devices. For instance, functionalized slotted-PC cavities with integrated microfluidics have been used in experiments to determine soluble avidin concentrations as low as 15 nM or 1 m/mL [146]. A LOD of less than 20 pM for anti-biotin, which equates to less than 4.5 fg of bound material on the sensor surface and fewer than 80 molecules in the modal volume of the integrated microcavity, has been experimentally proven to have extremely high efficiency [147]. For diagnosing malaria, a linear WG with a nanocavity-based 2D-PC-based biosensor has been suggested [148]. The changes in the transmission peak are studied at a wavelength of 1550 nm using a red blood cell (RBC) sample confined inside a nanocavity. A WG-based 2D-PC RR-based biosensor for diagnosing malaria has also been suggested. Utilizing a transmission peak at a wavelength of 2.07 microns, this sensor may identify infections [149].

From a technical perspective, PC-based photonic sensors such as integrated planar PCs and PC fibers are appropriate for multiplexing and label-free detection. For instance, large-scale chip-integrated PC sensor microarrays for biosensing on an SOI-based framework have previously been suggested and proven [142]. The creation of PCs often uses conventional and CMOS-compatible industrial techniques such as E-beam lithography, inductively coupled plasma (ICP) etching, and plasma-enhanced chemical vapor deposition (PECVD), enabling these sensors to be appropriate for mass-market and low-cost production. Ultimately, PC fibers may be produced quickly by stacking silica glass rods and tubes into a massive structure that has the desired pattern of holes. Since several poisonous gases (including CO_2_, CH_4_, and CO) display absorption lines in the mid-infrared wavelength region, gas sensors constructed on PC have been proposed [150]. A PC air-slot cavity-based high-precision gas index sensor with S = 510 nm/RIU has been suggested [151]. A high S = 3200 nm/RIU SPR nanocavity antenna array has also been suggested for gas sensing applications [152]. It is stated to have a guided-mode resonance gas sensor with an S = 748 nm/RIU [153]. A PC/Ag/graphene (Gr) architecture with an S of 1178.6 nm/RIU that functions as a RI sensor established on the Tamm state are suggested [154]. Some of the recent works on PC-based sensors are presented in Table 3.

**Figure 9:**
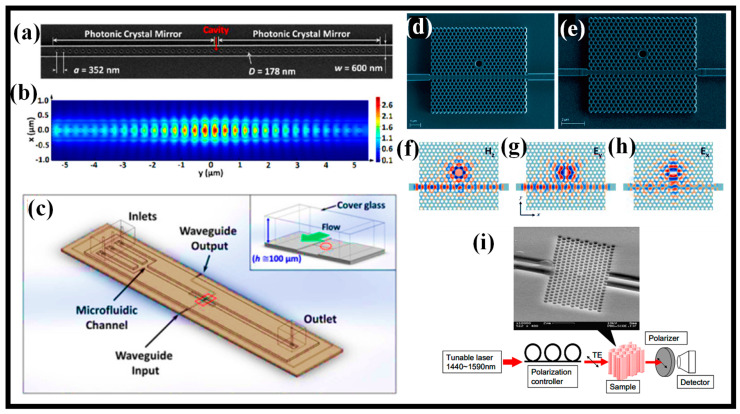
PC sensing devices, (**a**) SEM image of the PC resonator [169], (**b**) E-field distribution within the cavity [169], (**c**) 3D representation of an integrated optofluidic device [169], (**d**,**e**) electron micrograph images of the two PC sensor geometries, (**f**–**h**) E-field distribution at the resonant frequency of the optical mode [170], (**i**) SEM image of the fabricated sensing device and the graphic of the experimental setup [171].

It is suggested to use near-field optical trapping and light-scattering methods to analyze free-solution interactions between a single influenza virus and certain antibodies at the single particle level [169]. By examining how the virus’s Brownian fluctuations have changed, it is possible to determine how many antibodies are binding to an optically imprisoned influenza virus. The enlarged size of the virus brought on by antibodies attaching to the viral membrane is calculated using an analytical model. The stoichiometric values of anti-flu antibodies for binding to an H1N1 influenza virus are shown to be 26 ± 4 (6.8 ± 1.1 attogram). The nanophotonic tweezer can tackle molecules with a diameter of tens to thousands of nanometers, hence this method may be used for a variety of chemical interactions. The particle is optically trapped by using a PC cavity, as shown in Figure 9a [169]. Due to their powerful light confinement, PCs make an appealing sensing platform. PCs can be made to localize the E-field in the low RI region, which renders the sensors incredibly sensitive to a small RI change caused by bio-molecule immobilization on the pore walls. This contrasts with many sensing platforms that rely on the interaction between the small evanescent tail of the EM-field and the analyte. Point defects can draw defect states down from the air band or up from the substrate band when they are included in a PC. The resultant optical spectrum displays sharp transmission peaks inside the bandgap, and the exact locations of these peaks are governed by the pores’ refractive indices. The E-field confinement in the cavity is shown in Figure 9b [169]. The 3D model of the integrated optofluidic device is shown in Figure 9c [169].

Although the recognition-mediated detection of viruses or simulants under flow has been theorized, it has not yet been shown using 2D-slab PC sensors. A novel W1 WG-coupled 2D slab-PC sensor with a shape ideally suited to virus detection was designed and optimized in [170]. As this shape was estimated to create a transmission dip at the resonance wavelength that was neither too broad nor too shallow, the large-hole defects were placed either four rows or five rows distant from the W1 WG, as shown in Figure 9d and Figure 9e, respectively. The H-field and E-field distributions were taken at the resonant wavelength in the PC sensor, as shown in Figure 9f–h [170]. The sensor was proven to be capable of responding to the penetration of a single particle in both air and beneath an aqueous cover layer during proof-of-concept tests using fluorescent latex particles. The capability of the device to identify virus-sized particles under flow via a recognition-mediated mechanism was validated in further studies using antibody-functionalized sensors and viral simulants. The groundwork for the integration of 2D slab-PC sensors into fully integrated photonic sensor systems is laid out in [170].

A 2D-PC microcavity biosensor with extreme S is presented in [171]. The structure shown in Figure 9i is made up of a 400 nm-thick silicon slab segregated from the Si substrate by 1 micron of silica layer, which effectively confines the transmission modes vertically. The PC has a pore diameter of 270 nm and a lattice constant of 465 nm. The central pore diameter was decreased to 140 nm to reveal the defect. The sensor functioned close to its resonance at 1.58 microns and was constructed on an SOI wafer. Different amounts of resonance redshift are produced when proteins of various sizes are applied to the sensor’s interior surface. The current technology may detect a molecular monolayer with a total mass as tiny as 2.5 fg. The device’s functioning was confirmed by detecting the redshift associated with the binding of glutaraldehyde and bovine serum albumin. The theoretical predictions and ellipsometric measurements made on a flat oxidized silicon wafer surface are well-supported by the experimental findings [171].

**Figure 10:**
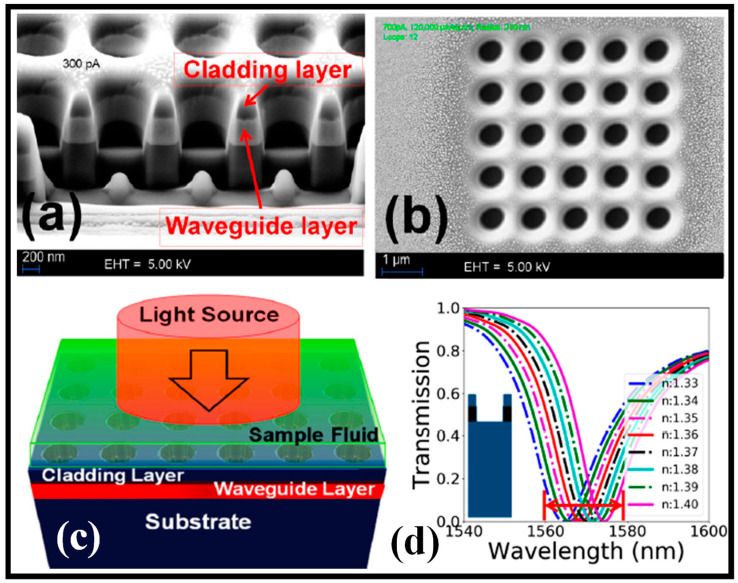
PC fluid sensor, (**a**) SEM image of the cross-sectional view of the PC structure [31], (**b**) SEM image of the top view of the PC structure [31], (**c**) numerical model of the sensing device [31], (**d**) transmission spectrum [31].

The fluid sensing application is presented for a dielectric PC device [31]. Focused ion-beam milling lithography was utilized to build the suggested nanosensor device, which was made of low-cost dielectric materials including SiO_2_ and Nb_2_O_5_. The instrument was evaluated quantitatively as a sensor for the range of biological refractive indices from 1.33 to 1.4. Following the manufacturing outcomes, the performance aspects of the biosensor device were investigated for 12 alternative structural profiles. It was demonstrated that the angular-wall profile of the manufactured structures degrades the sensor’s performance and that the ideal value of hole depth should fall between 930 and 1500 nm to achieve optimal functioning. For the device’s ideal design, an S of 185.117 nm/RIU and a FOM of 9.7 were obtained. Due to its inert material features, reliable construction, and simple integration with fiber-optic setups, the device is advocated for several biosensing applications. Figure 10a,b shows the SEM images of the cross-section and top view of the manufactured device. Figure 10c depicts the numerical model of the sensing apparatus. As the RI of the upper cladding of the device increases, the transmission spectrum performs a redshift, as shown in Figure 10d [31].

## 5. Metasurface-Based Sensors

Due to their exceptional EM properties, metamaterials have generated great interest during the past 20 years [172,173,174,175]. Metamaterials such as negative-index media [176], zero-index materials [177], and ultra-high-index materials [178] are arrays of specially structured scattering components that have been systematically created. MS, the two-dimensional equivalent of metamaterials, is significantly simpler to create and use [179,180]. It can demonstrate the incredible ability to manipulate EM wavefronts, which is primarily brought about by the interaction of an EM wave with these meta-atom constructions and their functional configurations. It has been suggested that biosensing and chemical detection are two applications for metamaterial-based sensors, which have been studied in the optical and microwave domains. The S of the sensor will also be enhanced by making use of the plasmon-induced transparency [181,182,183].

Modern contemporary science and technology applications require the MS’s transient response; however, traditional MS has limitations on its functioning in terms of tunability and customization. Typical MSs offer static, predetermined optical functionalities that are typically controlled by the configuration, shape, and topology of meta-atoms. Scientists have recently been focusing on creating flexible and reconfigurable MSs, where the meta-atoms’ size, form, and placement may be tweaked or changed in response to outside signals [184]. In addition, a significant number of investigators are constantly working to access reprogrammable MS [185] and multi-purpose MSs [182,186].

**Figure 11:**
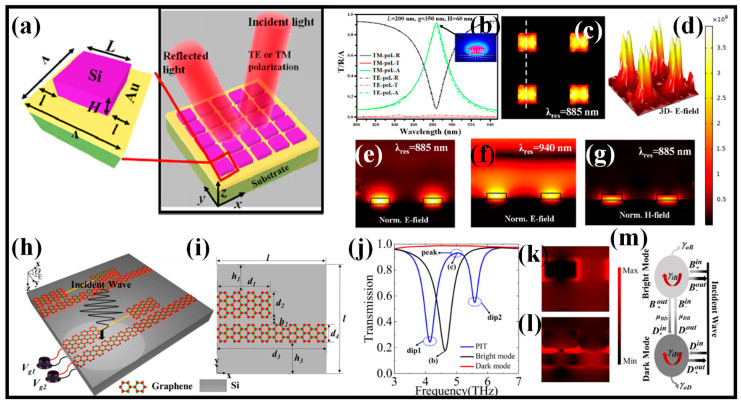
MS absorber designs, (**a**) HMSPA design [186], (**b**) T/R/A spectrum [186], (**c**) top view of the norm. E-field distribution [186], (**d**) 3D E-field distribution [186]. Cross-sectional view of the norm, (**e**) E-field distribution at the resonant wavelength [186], (**f**) E-field distribution at non-resonant wavelength [186], (**g**) H-field distribution at resonant wavelength [186], (**h**) graphical illustration of the tunable optical plasmonic Gr MS [187], (**i**) top view of the unit cell [187], (**j**) transmission spectrum [187], (**k**) E-field mapping at the dip for the bright mode [187], (**l**) E-field mapping at the peak for plasmonic-induced transparency [187], (**m**) theoretical coupled model [187].

In our earlier work, we thoroughly analyzed the sensing properties of a hybrid metasurface perfect absorber (HMSPA) established on square meta-atoms and hollow square meta-atoms [186]. Both designs are suitable for filtering operations since they can deliver >90% absorption in the narrowband area. In comparison to a square meta-atom, a HMSPA with a hollow square meta-atom is far more sensitive to minute changes in the RI of the surrounding medium, making it the perfect choice for biosensing applications. The hollow square meta-atom may increase the S of the square meta-atom-based HMSPA from 135 nm/RIU to 355 nm/RIU. Additionally, use of the suggested device for thermal sensing is made possible by placing a material that measures temperature on the surface of the MS. The hollow square meta-atom-based HMSPA has a temperature S of 0.18 nm/°C over the temperature range of 20 °C to 60 °C thanks to the exceptional thermo-optic coefficient of PDMS. With their simplicity in device manufacturing and strengths in light coupling, the suggested HMSPA constructions have the potential to be beneficial for filtering, biosensing, and temperature-sensing purposes [186]. The square meta-atom-based HMSPA and the TRA graph are illustrated in Figure 11a,b, respectively. The E-field and H-field distribution at the resonant wavelength is shown in Figure 11c–f and Figure 11g, respectively.

For terahertz detection and slow light purposes, a simple graphene MS with a continuous and truncated Gr strip was constructed and studied [187], as shown in Figure 11h,i. The findings show that destructive interference between bright and dark modes may result in plasmonic-induced transparency on the Gr MS. The Fermi level efficiently tunes the optical response’s transmission, reflectivity, and absorbance spectra. The polarization angle of the linearly polarized plane light is another factor that may be used to modify the plasmonic-induced transparency window. Interestingly, the suggested Gr MS exhibited interesting optical applications including sensing and slow light due to the field increase in the surface plasmon and significant dispersion. For the sensing attributes, the S, and figure of merit (FOM) may be up to 0.7928 THz/RIU and 8.12, respectively [187]. The suggested Gr-based MS might thus be anticipated to play a significant role in slow light devices and micro-nano optical sensing. In Figure 11j, the optical transmission pattern is shown as a black line when the Gr strip is shortened. The incident wave can effectively excite the truncated Gr strip, which can then function as a brilliant mode. When there is only a continuous Gr strip, the solid red line represents the optical transmission spectrum. The continuous Gr strip can function as a dark mode and cannot be effectively stimulated. When two Gr strips are present (Figure 11j), the solid blue line displays a plasmonic-induced transparency window. A peak is created in the bright mode’s spectrum thanks to the involvement of the dark mode, and two troughs are simultaneously produced in the plasmonic-induced transparency spectrum. Consequently, the plasmonic-induced transparency spectrum displays three transmittance bands. Under the excitation of the incident wave, the physical mechanism that causes the destructive interference effect is the interplay of two modes [187]. The E-field distribution diagram is then used to confirm that the analysis presented above is accurate. According to Figure 11k, the left and right sides of the truncated Gr strip have large concentrations of electrons, creating a powerful E-field. This shows that the incident wave immediately excites the truncated Gr strip. Many electrons are collected between two Gr strips in Figure 11l, which result in powerful E-fields. As a result, when the truncated Gr strip is inserted, the continuous Gr strip will be stimulated. Figure 11m describes the bright-dark mode resonant coupling theoretical paradigm [187]. Table 4 enlists the novel designs of the MS perfect absorbers suggested for sensing applications.

## 6. Plasmonic Sensors Based on Metal-Insulator-Metal Waveguide

One fascinating method used in integrated photonic sensors for the detection of chemical and biological species is surface plasmon resonance (SPR), which has been briefly discussed in [4]. Two distinct fundamental approaches have been proposed to implement optical sensing into planar WGs established on surface plasmon polaritons (SPP). The first necessitates the activation of a surface plasmon wave, while the second strategy entails the stimulation of “pure” plasmons [200]. In any event, the excited surface plasmon wave, or SPP, at the dielectric–metal interface is modified by a localized alteration of the RI close to the WG surface, which forms the basis of the sensing concept.

**Figure 12:**
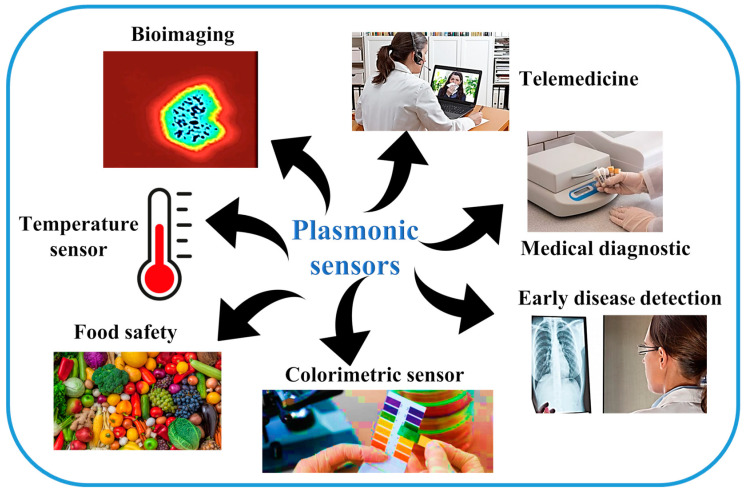
Applications of SPR sensors in telemedicine [212], medical diagnostic [213], early disease detection [214], colorimetric sensors [215], food safety, temperature sensors, and bioimaging [216].

From straightforward distance sensing to providing artificial vision for object identification, optical sensors are employed in a wide variety of applications [77]. The exploration of innovative nanostructures with custom functionality is one of the major problems that the current sensor industry faces [201,202]. The concept of using surface plasmon polaritons (SPPs) among other nanotechnologies distinguishes itself from its rivals [203]. Metallic nanostructures have the potential to generate and disperse EM radiation in completely unimaginable ways. SPPs are synchronized oscillations of free electrons at the metal/dielectric contact [204,205]. Plasmonic sensors have recently shown their benefits in several fields including chemical sensing [206], biological species [207], environmental monitoring [208], food safety [209], and medical diagnostics [210], thanks to the notable advancements achieved in micro- and nano-fabrication technology in recent years, as shown in Figure 12. These sensors are notable for their distinctive qualities in biochemical studies. A SPP-based test paper for the quick identification of COVID-19 has just been made available in Japan [211]. A unique coloration is visible when COVID-19 viruses are bound to antibiotic-coated gold nanoparticles that endure resonance peak shifts. Comparable techniques are frequently used in pregnancy tests.

A comparison of plasmonic sensors to those established on other platforms such as Si photonics or OF revealed that plasmonic sensors have a much smaller footprint and higher sensing capacities, making them very appealing and in high demand. With the assumption of achieving exceedingly integrated optical circuits due to their minor footprint, ease of incorporation, and good balance between light localization and transmission loss, SPP WG structures, mainly MIM WGs, have received much consideration due to their capacity to withstand the diffraction limit of light. With an S of 235 nm/RIU, a biosensing semiconductor nanowire RI sensor has been established [217]. Furthermore, the use of long-period fiber gratings as the foundation for OF RI sensors has been proposed [218]. According to Xu et al., the greatest experimental S for quasi-TM RRs is 135 nm/RIU [219]. By changing the WG thickness, it was possible to demonstrate a bulk S of 270 nm/RIU [220].

One of the fascinating subjects is sensing, and over the past years, numerous plasmonic sensing devices established on MIM WGs have been investigated numerically and proposed for use in temperature [221], gas [222], and RI sensing [223,224,225,226]. The suggested designs primarily support one function (either temperature or RI detection) at a time, despite the high S of these devices. Furthermore, because of their added intricate geometric elements, it is difficult to manufacture these patterns without leaving a few nanometers of error. To measure the RI, a plasmonic sensor was presented that combined a RR with circular tapered dots and a MIM WG with tapered dots [227]. The device’s S is around 1295 nm/RIU, but because of the complexity of its design, even a manufacturing mistake of a few nanometers can impair the function of the device. Another complex RI sensor was presented in [228] and consists of a MIM WG with two symmetric triangle stubs connected to a circular split-ring resonator cavity; the device has an S = 1500 nm/RIU [228]. Several parameters for this sensor arrangement must be carefully tuned to obtain the best sensing performance. With the suggested designs [229,230], a similar circumstance takes place. Although the numerical findings presented in this research look promising, the actual difficulties arise during the manufacturing stage of these devices when several factors must be tuned at a nanometer scale.

**Figure 13:**
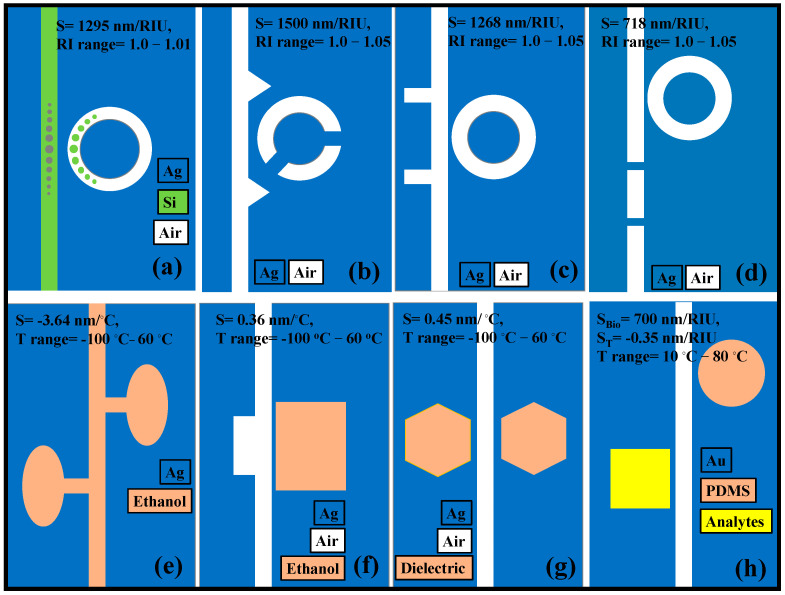
Graphic illustration (top view) of plasmonic sensors established on MIM WG, (**a**) RR linked to a MIM WG with tapered defects [227], (**b**) two triangle stubs paired with a split-ring nanocavity [228], (**c**) two stubs and a RR [229], (**d**) two baffles and a coupled ring cavity [230]. Thermal sensing devices, (**e**) ethanol-sealed asymmetric ellipse resonators [232], (**f**) ethanol-filled resonator cavity [234], (**g**) dual laterally side-coupled hexagonal cavities [235], (**h**) simultaneous temperature sensor and biosensor [231].

Recent research has shown that MIM plasmonic WG devices may be effectively used for temperature sensing applications when paired with thermal sensing media like ethanol or polydimethylsiloxane (PDMS) [231]. Zhu et al. suggested a sensor prototype with a very high S of −3.64 nm/°C that can only be utilized for temperature detection [232]. Additionally, Zhu et al. quantitatively examined a small Fano resonance temperature sensor using a sealed semi-square ring resonator made of PDMS. The sensors established on PDMS are extremely vulnerable to temperature changes because of their material’s high thermo-optic coefficient. Applications that require a high level of precision for temperature monitoring may benefit from the sensor. However, the cavity design is so complicated that at least five to six variables must be tuned to provide the greatest sensing performance. The S is around −4 nm/°C [233], which limits how flexible the manufacturing process may be. Using ethanol in a resonant cavity, Kong et al. suggested a temperature sensor with an S of 0.36 nm/°C [234]. In our previous work, a novel design of a plasmonic sensor was proposed for the simultaneous detection of temperature and analytes [231]. Figure 13 presents the different plasmonic MIM WG sensor designs for temperature and biosensing applications.

**Figure 14:**
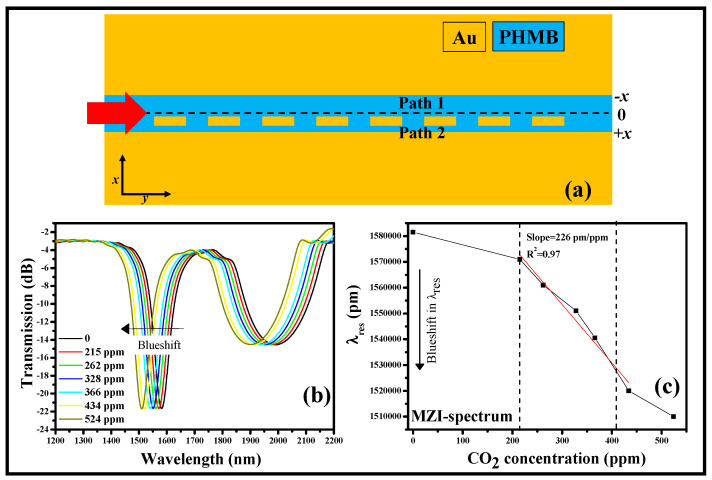
Modified plasmonic BG structure, (**a**) sensor design [236], (**b**) transmission spectrum [236], (**c**) S analysis [236].

A functional polymer called polyhexamethylene biguanide (PHMB) has a linear relationship with the CO_2_ level. As a result, it is conceivable to use this material to create CO_2_ gas sensors. By shifting the gold nano-blocks that are cyclically stacked in the MIM WG away from the line of symmetry, the plasmonic BG structure is asymmetrically changed [236], as shown in Figure 14a. Consequently, in conjunction with the broadband Bragg reflection, a narrowband MZI resonance dip also develops in the transmission continuum. Figure 14b shows that when the CO_2_ conc. rises from 0 ppm to 524 ppm, the MZI dip undergoes a blueshift. The recommended device’s CO_2_ S is 226 pm/ppm for the 215 ppm to 434 ppm range of gas concentrations, which is considerably greater than most of the previously proposed sensor designs. The CO_2_ gas conc. versus resonance wavelength graph is shown in Figure 14c. This paves the way for the realization of a single plasmonic sensor for multiple applications [236]. Table 5 highlights the noteworthy works related to MIM WG plasmonic sensors recently proposed for several remarkable applications.

## 7. Concluding Remarks and Outlook

Due to the growing need for sensing applications in industries including health care, defense, security, automotive, aerospace, the environment, and food quality control, to mention a few, photonic sensors have seen significant advancement in the last few decades [90,91,242]. Concerning the CMOS-compatible SOI technology, the development, and integration of microfluidic and photonic innovation and technology for the improvement of sensing performance in terms of sensitivity, the limit of detection (LOD), and detection multiplexing potential have been studied. Over the past few decades, photonic sensors have been the focus of many studies, particularly with regard to the detection of a wide range of biological and chemical substances. In this regard, photonic lab-on-a-chip systems offer cutting-edge photonic sensing because they are anticipated to have higher sensitivity and selectivity in addition to high stability, immunity to EM interference, and product advancements such as relatively small assimilation scales and lower costs. In this review, recent advances in numerous sensing technologies such as optical WG-based sensors, optical fiber-based sensors, photonic crystal-based sensors, metasurface-based sensors, and MIM WG-based-plasmonic sensors were extensively presented.

There are several uses for WG-based optical sensors including the label-free detection of chemical or biological analytes that precisely attach to functionalized WG surfaces. By leveraging well-known photonic integration platforms like silicon or silicon nitride, these sensors show significant prospects for downsizing and economical mass manufacturing. The most popular types of sensor configurations are established on interferometers such as those in the Mach–Zehnder and Young configuration [74], or on resonant components including ring, disk, and Bragg resonators, which may be improved even further by making use of the Vernier effect [62]. These sensor designs, which enable long effective contact durations with the analyte and combine high sensitivity with a compact device footprint, are well-suited for high-density integration into massive parallel arrays. Various methods for optimizing certain kinds of WG have been reported in recent years for both surface sensing and the detection of bulk changes in RI in the WG cladding (homogeneous sensing). These studies, however, frequently focus on a few WG types and geometries on a small number of material platforms such as silicon [2], silicon nitride (Si_3_N_4_) [34], and polymers [74]. Therefore, the greatest surface sensitivities across various WG types and integration platforms cannot be compared generically. Additionally, the majority of sensitivity assessments only take into account specified surface layers with predetermined refractive indices.

EM interference and radio frequency interference do not effect OF-based sensors. It is secure and suitable for use in harsh situations with high vibration. It is tolerant of corrosive conditions and high temperatures. Due to its high sensitivity, even minor variations in the ambient medium may be observed [80,81,82,83]. Its size and weight are both small and manageable. A large bandwidth and a broad dynamic range are provided. Both multiplexing and remote sensing functions are available. It has several sensing applications including mechanical measurement, electric measurement, magnetic measurement, chemical and biological sensing, among others. Nearly all physical measurements including temperature, pressure, flow, liquid level, displacement, vibration, rotation, magnetic and electric fields, and acceleration can be determined.

MSs are now considered as emerging study areas because of their peculiar and highly controllable light scattering in ultracompact volume characteristics. The geometric dimensions of each meta-atom, a key MS component, and their subwavelength spacing determine how well MSs perform [181,182,183]. Meta-atoms are made up of one or more subwavelength nanostructures made of high-index dielectrics or noble metals. They are designed to display the appropriate effective local optical responses, which may be described in terms of electric and magnetic polarizabilities as well as amplitude and phase. Exotic functions with several possible uses including transmission, virtually perfect absorption, and negative RI have been identified. RI bio-sensing is the most practical and illustrative of the potential uses, which also include superlens, slow light, and cloaking devices. Changes in refractive indices are caused by bio-molecular interactions in the analyte layers [189]. To be an essential component of diverse chemical and biological sensing technologies, the RI sensor must provide distinct capabilities for sensitive and label-free biochemical experiments [188].

PCs offer an exciting method for achieving excellence in sensing applications. Since many photonic designs have been extensively studied and used in photonic sensing, PCs show the strong optical confinement of light to a very tiny volume, permitting the detection of chemical species with nanometer-scale dimensions. Additionally, very effective ultra-compact sensor chips may be produced by integrating modern chemical surface functionalization processes with microfluidic devices. From a technical perspective, PC-based photonic sensors such as integrated planar PCs and PCFs are appropriate for multiplexing and label-free detection. For instance, large-scale chip-integrated PC sensor microarrays for biosensing on an SOI-based framework have recently been suggested and proven [142]. PCs are typically manufactured using conventional and CMOS-compatible technical techniques such as E-beam lithography, ICP etching, and PECVD, rendering these sensors appropriate for mass-market and low-cost manufacturing. Ultimately, PCFs may be produced quickly by stacking silica glass rods and tubes into a huge structure that has the desired pattern of holes.

Because of its adaptable on-chip inclusion, little bending loss, increased propagation length, subwavelength confinement, and relative simplicity of manufacturing, plasmonic MIM WGs formed on SPPs have been thoroughly explored as a potential area of optical WGs [227]. Due to the demand for ultra-high sensitive biological sensors, plasmonic RI sensors produced on MIM WGs have attracted a lot of attention [4]. Compared to more conventional approaches like fluorescence analysis, sensors established on the SPP phenomenon are more analyte-compliant and do not need additional processing steps such as labeling [209]. The use of SPPs has also attracted significant consideration in the field of optical sensing since its initial gas sensing demonstration. Plasmonic sensors made possible by MIM WGs may be used for a variety of tasks including the detection of temperature, pressure, and RI. There are several applications for RI sensors in the biological sciences. For instance, monitoring changes in the RI makes it possible to determine the solution conc. and pH level [239,250].

Describing the technologies of optical sensor systems, one cannot but briefly mention Raman spectroscopy. This is a vast topic that deserves a separate article. However, we will briefly dwell on this topic in the current work. Raman spectroscopy is one of the most common spectroscopy technologies in biosensors today [253]. Raman scattering is a non-elastic phenomenon in which incoming photons either absorb energy from or release energy to the vibrational and rotational movements of a target molecule. Consequently, the Raman spectra generated contain bands that are specific to the molecular structure, thereby producing distinct chemical signatures unique to each molecule [254,255].

SERS has become very widespread as a method of tissue analysis in diagnosing various diseases including cancer [256] and intraoperative diagnostics [257]. For example, Raman systems have shown promising results in distinguishing between cancerous and healthy brain tissue with high accuracy. In [258], a hand-held optic Raman probe and a 785 nm NIR Laser were utilized to achieve a sensitivity of 93% and a specificity of 91% for the brain tissue analysis. In the study of pancreatic cancer, the authors, using the Raman system, detected the MUC4 biomarker at a wavelength of 632.8 nm [259]. Further studies [260] when detecting CA19-9, MMP7, and MUC4 markers characteristic of pancreatic cancer using SERS at a wavelength of 785 nm showed the great potential of the method for early diagnosis of the disease. Raman spectroscopy can also be successfully used for viral diagnostics. In [261], the authors provide an example of the successful detection of common viruses including the SARS-CoV-2 coronavirus.

Raman spectroscopy is successfully used to analyze biological processes occurring in plant tissues [262,263]. Thus, [264] illustrated the possibility of using Raman spectroscopy to determine melon seeds infected with the bacterium *Acidovorax citrulli*. The authors of [265] showed that by using Raman spectroscopy, it is possible to achieve high (up to 82.8%) accuracy in determining the damage to tomatoes by the bacterium Candidatus Liberibacter solanacearum (type B). It should be noted that the further development of the use of SERS is inextricably linked to the use of machine learning methods to improve the accuracy of diagnostics. In [266,267], the authors provide an overview of intelligent algorithms used to classify Raman spectrograms and identify diseases including analysis based on deep learning methods, binary classification, support vector machines, and various types of neural networks.

## Figures and Tables

**Table 2:** Recently proposed photonic sensors established on different types of OFs.

Ref.	Fiber Type	Application	Sensitivity	Sensing Mechanism	Year
[123]	U-shaped MMF	Biosensing	1251.44 nm/RIU	LSPR	2020
[124]	Plastic OF	Cholesterol detection	140 mg/dL to 250 nm/dL	-	2017
[125]	SMF	Temperature	210.25 KHz/°C	Vernier effect	2020
[126]	Fiber tip integrated ZnO-nanowire-nanograting	Temperature	0.066 nW/°C	Bragg reflection	2023
[127]	Magnetic field micro-nano fiber	Magnetic field	69 pm/Gs	Mach-Zehnder interference	2022
[128]	PC fiber	Biosensing	12,000 nm/RIU and 16,000 nm/RIU	SPR	2020
[129]	D-shaped OF	Biosensing	5161 nm/RIU	SPR	2018
[130]	D-shaped OF	Biosensing	4122 nm/RIU	LMR	2018
[131]	PC fiber	Temperature	0.1636 nm/°C	Quantum dot	2009
[132]	D-shaped PC fiber	Biosensing	20,000 nm/RIU	SPR	
[133]	D-shaped PC fiber	Biosensing	21,700 nm/RIU	SPR	2017
[134]	Octagonal PC fiber	Transformer oil	(I) 31,240 RIU^−1^ (x-pol.), (II) 30,830 RIU^−1^ (y-pol.)	Plasmonic	2020
[135]	Elliptical channel PC fiber	Malaria detection	11,428.57 nm/RIU, 9473.68 nm/RIU, 9655.17 nm/RIU	-	2021

**Table 3:** Biosensors established on PC reported in recent years.

Polarization	RI Range	S (nm/RIU)	Reference
(I) x-polarized mode (II) y-polarized mode	-	4156.82 (I) 3703.64 (II)	[155]
y-polarized mode	1.36–1.40	33,500	[156]
(I) x-polarized mode (II) y-polarized mode	1.33–1.45	10,448.5 (I) 8230.07 (II)	[157]
(I) x-polarized mode (II) y-polarized mode	1.330–1.370	5000 (I) 10,000 (II)	[158]
(I) x-polarized mode (II) y-polarized mode	1.33–1.40	9000 (I) 9000 (II)	[159]
x-polarized mode	1.0–1.05	508	[160]
x-polarized mode	-	510	[151]
y-polarized mode	1.0–1.0010	3200	[152]
y-polarized mode	1.33–1.43	2150	[161]
y-polarized mode	1.33–1.37	1000	[162]
y-polarized mode	1.4–1.44	9180	[163]
(I) x-polarized mode (II) y-polarized mode	1.33–1.34	2000 (I) 1700 (II)	[164]
x-polarized mode	1.0–1.377	160	[72]
x-polarized mode	1.0–2.0	65.7	[165]
y-polarized mode	1.0–1.8	396	[166]
y-polarized mode	1.0–1.33	300	[167]
-	-	10,000–12,857 for different cancer cells	[168]

**Table 4:** Recent advances in narrowband MS perfect absorbers for sensing applications.

Ref.	Device Design	Material	Wavelength Range	Application	Sensitivity
[186]	Square and hollow square meta-atoms	Si-dielectric-metal	NIR	(I) Temperature (II) Biosensing	(I) −0.18 nm/°C (II) 355 nm/RIU
[188]	Nano-trench	Graphene	NIR	Biosensing	500 nm/RIU to 1000 nm/RIU
[189]	Rectangular strip	Dielectric-metal	MIR	Biosensing	1800 nm/RIU
[190]	Square	Si-dielectric-metal	NIR	Biosensing	460 nm/RIU to 492 nm/RIU
[191]	Square	Metal-dielectric	NIR	Biosensing	840 nm/RIU
[192]	Cylinder	InSb	THz	Biosensing	1800 GHz/RIU to 1900 GHz/RIU
[193]	Cylinder	Metal-dielectric-metal	NIR	Biosensing	1109 nm/RIU to 1290 nm/RIU
[194]	C-shape split ring	GST phase changing material	NIR	Biosensing	1000 nm/RIU
[195]	Metal disc	Metal-graphene	FIR	Biosensing	3.98 μm/RIU to 5.06 μm/RIU
[182]	Cylinder	Si-dielectric-metal	NIR	Gas	17.3 pm/ppm
[196]	Vertical strip-ring	Metal	THz	Biosensing	908 nm/RIU to 4367 nm/RIU
[197]	-	Gold-Si-Graphene	THz	Biosensing	66 GHz/RIU
[198]	Cross array	Si-gold	THz	Biosensing	25.3 THz/RIU to 41.3 THz/RIU
[199]	Square	MoS_2_-TiO_2_ on SiO_2_	680–720 nm	Biosensing	222 nm/RIU

**Table 5:** Recently proposed highly attractive plasmonic sensors established on MIM WG.

Ref.	Application	Sensitivity	FOM	Q-Factor	Year
[237]	(I) Gas (II) Biochemical	(I) 3639.79 nm/RIU, (II) 7530.49 nm/RIU	91.02	99.75	2022
[236]	Gas	226 pm/ppm	0.004	24.7	2022
[238]	Bio-analytes	3000 nm/RIU	9.7 × 10^5^	-	2022
[231]	(I) Bio-analytes (II) Temperature	(I) 700 nm/RIU, (II) −0.35 nm/°C	(I) 21.9, (II) 0.008	-	2021
[239]	(I) Bio-analytes (II) Temperature	(I) −0.58 nm/°C and −0.64 nm/°C (II) 1240 nm/RIU and 1350 nm/RIU	(I) 8.6 and 1955.2, (II) 18.74 and 691	-	2022
[240]	Bio-analytes	1578 nm/RIU	175	-	2022
[241]	(I) Temperature (II) Glucose	(I) 1.55 nm/°C, (II) 4074 nm/RIU	2.45 × 10^−6^	-	2022
[242]	Bio-analytes	825.7 nm/RIU	13.14	-	2022
[243]	Bio-analytes	7872 nm/RIU	394	-	2022
[244]	Pressure	10.96 and 10.5 nm/MPa	-	-	2022
[245]	Bio-analytes	2473 nm/RIU	34.18	56.35	2021
[246]	Pressure	25.4 nm/MPa	-	-	2021
[247]	Pressure	16.5 nm/MPa	-	-	2018
[248]	Bio-analytes	1948.67 nm/RIU	29.52	29.90	2020
[249]	Bio-analytes	2300 nm/RIU	31.5	31.1	2020
[222]	Gas	135.95 pm/ppm	-	-	2021
[250]	Bio-analytes	793.3 nm/RIU	52.9	82.1	2019
[251]	(I) Bio-analytes (II) Temperature	(I) 1406.25 nm/RIU (II) 0.45 nm/°C	156.25	-	2021
[252]	(I) Temperature (II) Glucose	(I) 1.525 nm/°C (II) 0.45 nm·L/g	52.73	-	2021

## Data Availability

Not applicable.

## Abbreviations

Waveguide = WG; Electromagnetic = EM; Silicon on Insulator = SOI; Surface plasmon resonance = SPR; Surface plasmon polariton = SPP; Ring resonator = RR; Polydimethylsiloxane = PDMS; Metal-insulator-metal = MIM; Photonic crystal = PC; Refractive index = RI; Sensitivity = S; Limit of detection = LOD; Plasma enhanced chemical vapor deposition = PECVD; Inductively coupled plasma = ICP; Photoplethysmography = PPG; Polyhexamethylene biguanide = PHMB; Single mode fiber = SMF; Multimode fiber = MMF; Photonic crystal fiber = PCF; Metasurface = MS; Figure of Merit = FOM; Graphene = Gr; Bragg grating = BG; SERS = surface-enhanced Raman spectroscopy.
